# Endoscopic Vacuum Therapy for Treating an Esophago-Pulmonary Fistula after Esophagectomy: A Case Report and Review of the Literature

**DOI:** 10.1159/000529725

**Published:** 2023-02-23

**Authors:** Imad Kamaleddine, Magdalena Popova, Ahmad Alwali, Clemens Schafmayer

**Affiliations:** General, Visceral, Thorax, Vascular and Transplantation Surgery, Rostock University Medical Center, Rostock, Germany

**Keywords:** Esophago-pulmonary fistula, Endoscopic vacuum therapy, Anastomotic leak, Endoscopy, Surgical complications, Esophageal cancer, Case report

## Abstract

An acquired esophago-respiratory fistula represents an abnormal connection between the esophagus and the respiratory system. It is usually caused by malignancy and infection, or it occurs as a complication after surgery or radiation therapy. It can be divided according to its anatomical level into esophago-tracheal fistula, esophago-bronchial fistula, and in the rarest case, esophago-pulmonary fistula (EPF). We present a case of EPF aggravating an anastomotic leak (AL) after the Ivor-Lewis operation for esophageal cancer. The leak was treated with endoscopic vacuum therapy (EVT) using the Eso-Sponge® system (B. Braun Melsungen AG, Melsungen, Germany). In the further course of treatment, an EPF was suspected by a new onset of severe cough after oral fluid intake. The suspicion was confirmed by injecting methylene blue dye into the paraesophageal-extraluminal cavity during endoscopy and attesting to its presence in the respiratory tract by simultaneous bronchoscopy. Furthermore, an oral contrast computed tomography scan showed the presence of contrast in the right lower lobe of the lung. This complication was treated conservatively with EVT and antibiotics. Nutrition was administered through the existing jejunostomy. Both fistulas and the paraesophageal cavity were fully healed, oral intake was maintained, and the patient was discharged. This rare life-threatening complication can be treated conservatively. Its management is challenging, controversial, and lacks a general consensus.

## Introduction

Esophagectomy with two-field lymphadenectomy remains the standard of care for patients with esophageal cancer. Despite the technical advances of surgical procedures, this procedure is still associated with high rates of morbidity and mortality, with the anastomotic leak (AL) alone affecting between 2 and 15.9% of the patients [1]. Once an abnormal connection is established between the digestive and respiratory system, there occurs what is called an acquired “esophago-respiratory fistula.” It is classified depending on its location as esophago-tracheal fistula, esophago-bronchial fistula, and more rarely esophago-pulmonary fistula (EPF) [2]. Although AL treatments are well described using endoscopic methods [3], there is a lot of uncertainty surrounding the management of esophago-respiratory fistula [2]. In this paper, we present the case of an AL aggravated by the rare EPF after an Ivor-Lewis operation for esophageal cancer, in accordance with CARE Checklist.

## Case Report

**Fig. 1:**
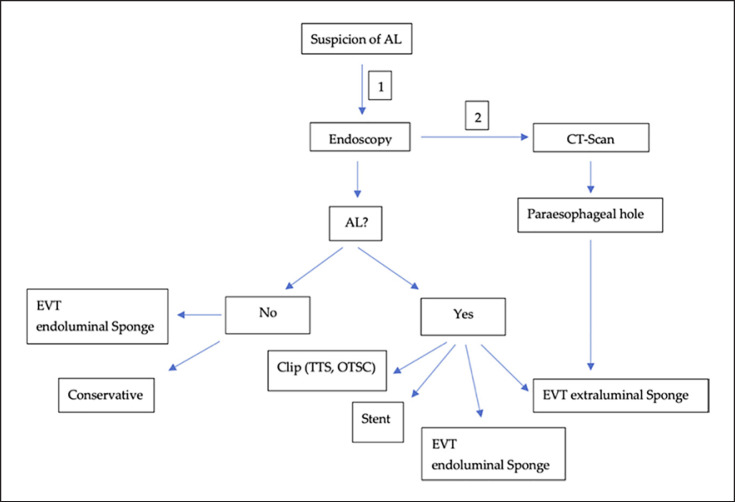
Algorithm illustrating our protocol of AL management after esophagectomy.

**Fig. 2:**
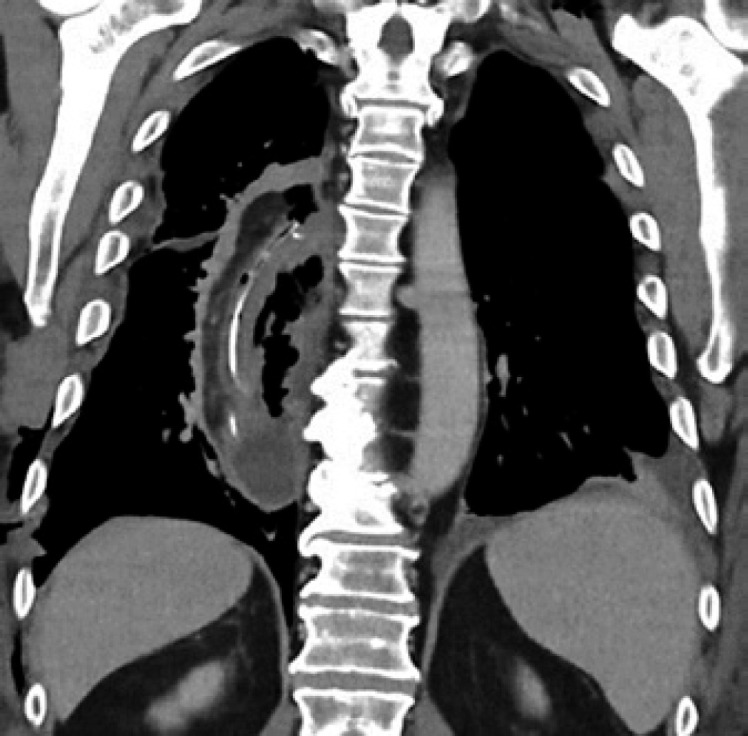
Sponge in situ after diagnosing the AL on the 8th POD.

**Fig. 3:**
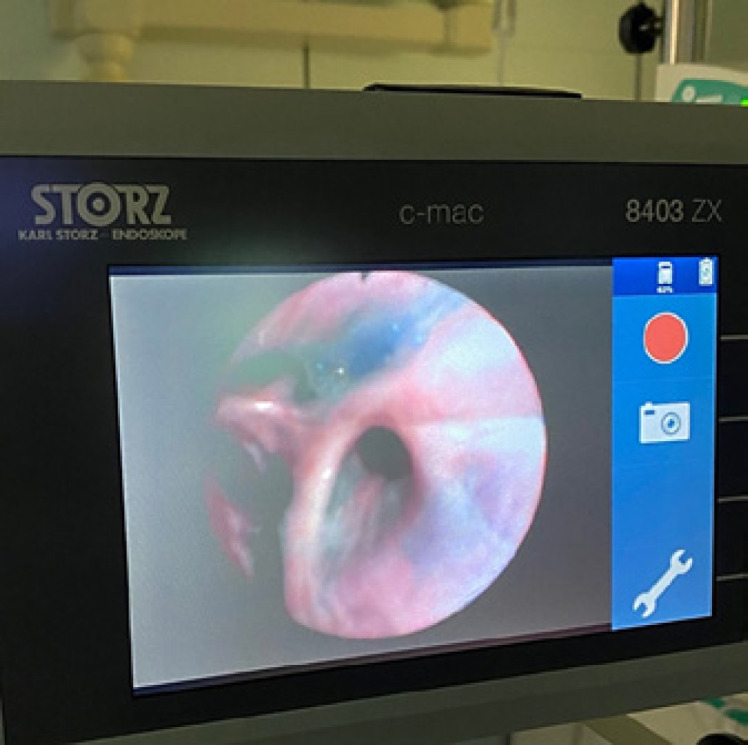
CT scan with oral contrast showing the EPF with intrapulmonary contrast.

**Fig. 4:**
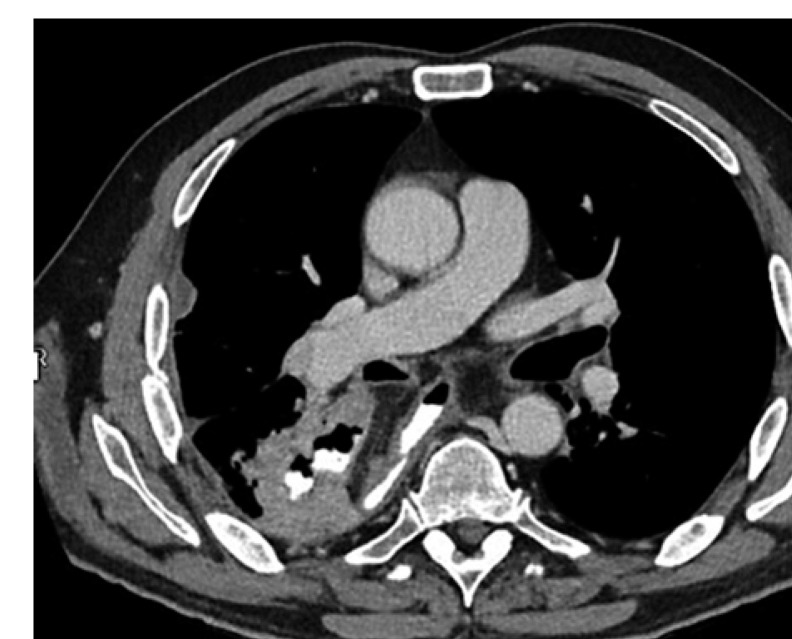
Methylene blue dye in the bronchial system.

**Fig. 5:**
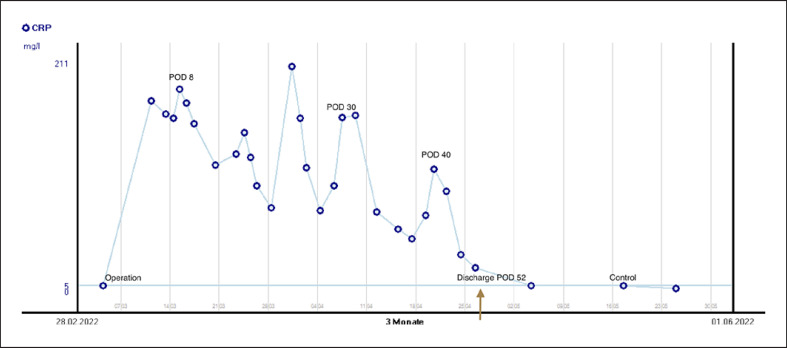
CRP level's chart. CRP, C-reactive protein.

**Fig. 6:**
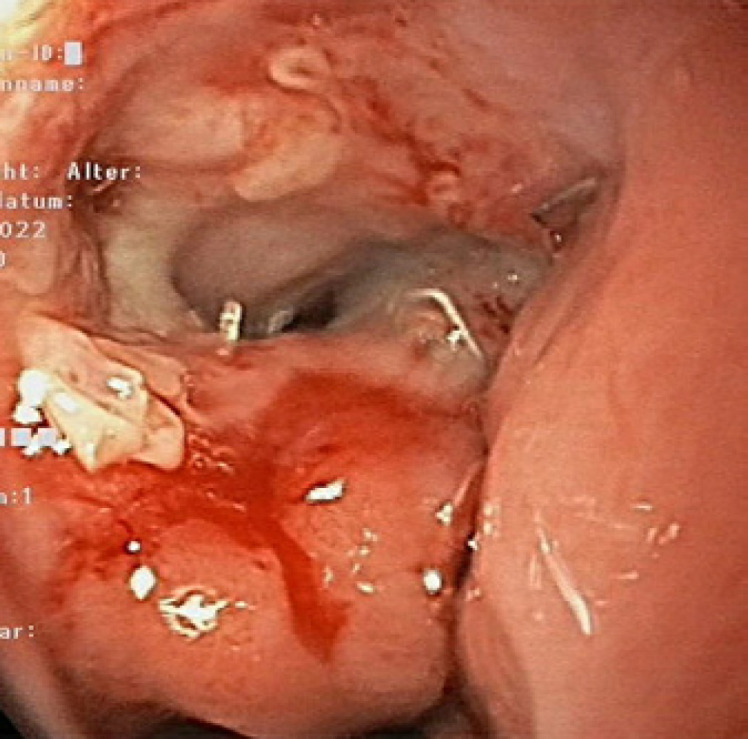
Gastroscopy on the 51st POD.

**Fig. 7:**
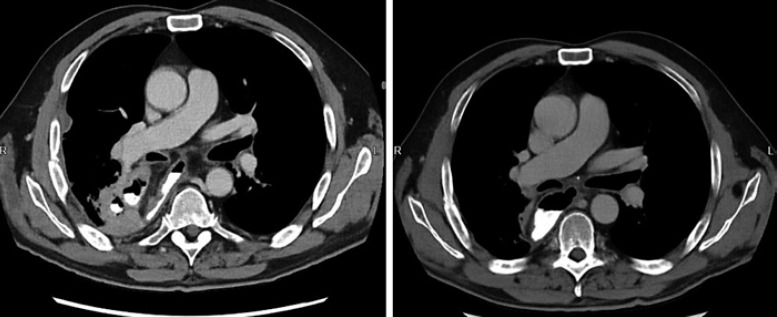
Comparable views of CT scan before (left) and after (right) the EPF closure.

**Fig. 8:**
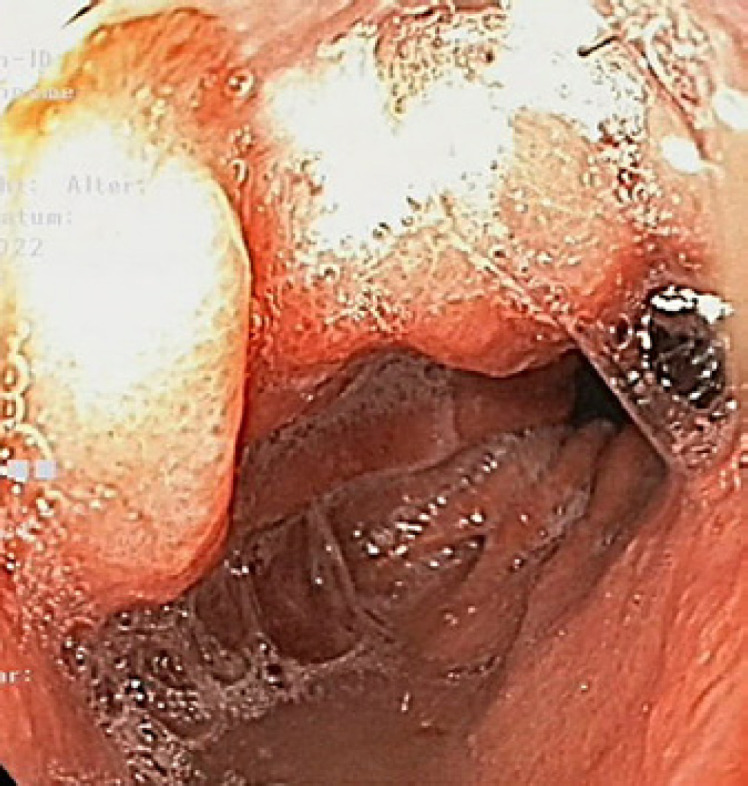
Gastroscopy 2 weeks after discharge showing complete closure of the cavity.

A 59-year-old Caucasian male (BMI: 31.6) with stage IIb T2 N+ adenocarcinoma of the lower esophagus underwent Ivor-Lewis-Esophagectomy after neoadjuvant chemotherapy using the FLOT protocol. He had a history of arterial hypertension and prostate hyperplasia without any other significant secondary diagnosis. His family, genetic, social, and drug histories were not significant. After obtaining informed consent, we proceeded with the operation. Our standard procedure includes, in addition to esophageal resection with lymphadenectomy, the construction of a neoesophagus using a stomach conduit, which was anastomosed to the proximal esophageal stump in an end-to-end fashion using an ECHELON CIRCULAR^TM^ powered stapler 25 mm (ECP, Ethicon Endo-Surgery, Inc., Cincinnati, OH, USA) and added a handsewn second layer of reinforcement with absorbable PDS 4/0 sutures. Insertion of a jejunal feeding tube and cholecystectomy are also a typical part of the operation. Three drains are inserted: a Robinson drain placed in the subhepatic area, exiting in the right upper quadrant of the abdomen, and two Easy-Flow drains attached to the proximal part of the stomach at the level of the hiatus. During the thoracic part of the operation, those are pulled through to the thorax with the stomach conduit and fixed at the anastomotic level using a resorbable suture. These drains are attached to the skin in the left upper quadrant of the abdomen. The operation was uneventful. The postoperative course was smooth until the 8th postoperative day (POD) when the Easy-Flow drains showed turbid secretion. Although the patient did not have clinical signs of infection, bloodwork on the same day showed a peak of C-reactive protein reaching 187 mg/L. Following our protocol (Fig. 1) [4], an upper gastrointestinal tract endoscopy was performed under sedation with Propofol. It showed the presence of a fibrin coat at the anastomotic level with an indirect sign of AL when the Easy-Flow drain bag was filled with air. We began the endoscopic vacuum therapy (EVT) using an Eso-Sponge® (B. Braun Melsungen AG, Melsungen, Germany). The Eso-Sponge® is a CE-certified product that allows a standardized application of an adequate treatment regime. During endoscopy, the paraesophageal hole, when found, is intubated. A plastic overtube is pushed under visual control until the end of the cavernous hole. Using a pusher, a size-adjusted polyurethane sponge is inserted into the paraesophageal cavernous hole. The flexible tube attached to the sponge is placed transnasally. Through it, a constant negative pressure of approximately 125 mm Hg is applied by a vacuum pump [5, 6, 7, 8]. In our case, the first sponge was placed intraluminaly at the anastomosis level. Thorax/abdomen computed tomography (CT) scan with intravenous (iv) contrast was performed and ruled out the presence of associated mediastinitis (Fig. 2). Furthermore, we started iv antibiotic therapy as follows: Meropenem 1 g 2 times daily, Vancomycin 1 g 2 times daily and Caspofungin 70 mg 2 times daily. This antibiotic course continued for 2 weeks. Typically, an endoscopic change of the EVT sponge is performed every 2–3 days, allowing visual assessment of the AL and the evolution of treatment. The second change of the sponge confirmed the presence of a small defect in the anastomosis. This defect was endoscopically balloon-dilated, allowing the drainage of the paraesophageal hole and thus the extraluminal placement of the new sponge. From this day on, the patient was allowed to have clear oral fluids. The treatment with Eso-Sponge® continued for 21 days until the 29th POD changing in total 8 sponges. During this period, the insufficiency hole showed a very good tendency of granulation tissue growth. On 30th POD, the patient developed a new onset of cough, which was aggravated by a supine position or oral intake. This prompted suspicion of a pulmonary complication. A new oral contrast-guided thorax CT scan revealed the presence of an acquired EPF (Fig. 3). Subsequent simultaneous gastroscopy with bronchoscopy under general anesthesia was performed. The existence of communication between the two organs was confirmed by intrabronchial visualization of the methylene blue dye, which was injected primarily through the gastroscope into the paraesophageal insufficiency hole (Fig. 4). In its granulation tissue, a fistula ostium could not be located. Although the patient maintained a stable respiratory status, he was transferred to the intensive care unit for constant monitoring. His oxygen saturation did not drop below 92% in room air. Due to the positive tendency of granulation and the patient's stable hemodynamic situation, we decided against a surgical intervention and proceeded with conservative management. This consisted of a new episode of antibiotic therapy using the above-mentioned medications while continuing the EVT. At this point, the paraesophageal insufficiency hole measured 14 cm in depth (online suppl. Video 1; see www.karger.com/doi/10.1159/000529725 for all online suppl. material). Nutrition proceeded through the jejunostomy catheter. Three new Eso-Sponge® changes were performed over 10 days. On the 40th POD, the coughing episodes ceased, allowing the EVT to be discontinued (online suppl. Video 2). After the gradual restoration of nutrition, a normal oral diet could be reached on the 46th POD. Bloodwork showed a regressive level of C-reactive protein (Fig. 5). A control gastroscopy performed on the 51st POD revealed a very small opening leading to the insufficiency hole without clinical relevance (Fig. 6). The patient was clinically stable and was able to be discharged on the 52nd POD. He returned 2 weeks later for a planned ambulatory CT scan and gastroscopy. Both showed a completely healed EPF (Fig. 7, 8). During his homestay, the patient did not experience any episodes of cough or dysphagia. He gained 2 kg in comparison with his prior weight at the time of discharge. The enteral feeding tube was then removed.

## Discussion

In reviewing the literature, we observed three pitfalls concerning the management of this life-threatening complication. First, the ambiguity of the nomenclature such as the use of all the following terms to describe the same problem: tracheo-esophageal or broncho-esophageal fistula [9], airway gastric fistula [10], esophago-alveolar and esophago-pulmonary fistula [11]. Second, most of the published articles were case reports or retrospective studies of small groups of patients. Including the consensus of Chinese experts published in 2018, no other international consensus was found [11]. Finally, EPF was never described as a postoperative complication but only in the context of direct tumor infiltration into the pulmonary parenchyma [12, 13]. Even in this case, it represented the rarest type of fistula, accounting for 3–11% [14]. Symptoms range from nonspecific cough to severe sepsis associated with mediastinitis and occur typically after oral intake. The diagnosis is made endoscopically (simultaneous bronchoscopy and gastroscopy) with the use of methylene blue dye [15] or by CT scan [12]. The success of treatment depends on the type of fistula, its width, the patient's pulmonary status, and the experience of the center. Treatment options include endoscopic stenting: bronchial, esophageal, or both (double stenting) [11], the use of Over-The-Scope-Clip OTSC® (Ovesco Endoscopy GmbH, Tübingen, Germany) [16], the use of cardiac septal defect occluders [17] or surgery. In our particular case, we were dealing with an AL following the Ivor-Lewis operation. This AL was treated using the well-established EVT. The vacuum administered through the sponge leads to a build-up of granulation tissue, resulting in faster cleaning and complete healing of the wound [18, 19]. Every 3 days, the sponge was exchanged endoscopically. An extension of the intervals may lead to an increased frequency of sponge growth, therefore causing a challenging endoscopic intervention, putting the patient at higher risk with less benefit [20]. The repeated EVT changes allow both lavage and inspection of the lesion to be performed over a period of time. Recent literature shows that EVT is clearly the most effective method to treat perforations and upper gastrointestinal tract leaks, with high success rates ranging from 76 to 91% [21]. Another option in treating the AL after esophagectomy is the new VACStent® (VAC Stent Medtec AG, Steinhausen, Switzerland) [22]. This novel hybrid device comprises a fully covered stent and a polyurethane sponge cylinder connected to a vacuum pump, allowing for the benefits of both stenting and EVT. In our case, this was not an option because of the large paraesophageal hole's length.

One of our main questions was, “Why this particular case of AL was complicated by an acquired EPF?” Two factors might have led to this:

- The paraesophageal insufficiency hole was unusually long and measured approximately 14 cm, therefore explaining the need for an extended treatment period (23 days and 8 sponge changes).
- The ongoing inflammation is in a very vulnerable location, directly at the level of the peripheral lung parenchyma.

Irradiation can also contribute to tissue's fragility, which was not the case in our patient [23]. Among the other possible factors, an iatrogenic etiology might have occurred. Although the EVT is performed with too much care and minimal force, the sponge is inserted through the overtube and then pushed blindly into the cavity. This part of the technique might cause a slight injury to the surrounding, inflamed, and vulnerable tissue. However, this is hard to confirm or exclude. The treatment approach was intricate. Due to the distal injury to the bronchial system, a bronchial stent was out of the question. The use of an esophageal stent and or VACStent®(VAC Stent Medtec AG, Steinhausen, Switzerland) was feasible, but it would leave a huge undrained paraesophageal insufficiency hole behind risking the subsequent formation of a lung abscess and thus leading to sepsis. Among other ideas, we discussed the injection of platelet-rich plasma (PRP) into the suspected area of the fistula as an attempted supportive therapy. This treatment has shown potential benefits for certain clinical situations in the wound healing process. However, it lacks standardization of the preparation, was never used before in the digestive system, and more clinical research need to be performed before being implemented as first-line therapy [24]. Additionally, this method will also need a concomitant therapy. Two other options were still on the table:

1. Continue using the EVT and start deep from the base of the hole, hoping that the granulation tissue will overcome the microscopic fistula tract.
2. Perform a surgical intervention through re-thoracotomy on the 30th POD.

The patient was hemodynamically stable with an acceptable respiratory status, and taking into account the risk of ending the procedure with a right lower lobectomy and the associated high mortality of a reintervention [25], we decided to proceed with EVT. This therapy was found to have a positive outcome in patients treated for AL and concomitant septicemia and mediastinitis [18, 19]. When compared to endoscopic stenting, the EVT showed superior results in terms of patient survival (80–96%) [8, 26, 27, 28, 29] and complete wound cavity healing in 84–100% of cases [30, 31, 32, 33, 34, 35]. Although −125 mm Hg is the standard negative pressure used in the practice of a lot of high-volume centers, a lower pressure setting might be a reasonable step when the EVT sponge is placed in close proximity to chest organs [36]. One long-term complication after vacuum therapy is the possible excessive stenosis as a result of scarring. This can be reversible by endoscopic dilatation [8, 26, 29, 37, 38]. This particular complication occurred in our case 4 months after the end of treatment and was successfully treated with two endoscopic balloon dilatations in a 2-week interval. Afterward, the patient was free of complaints. To our knowledge, this is the first time an EVT has been used exclusively to successfully treat acquired EPF.

## Conclusion

An acquired EPF is an uncommon complication that aggravates an AL following an esophagectomy for cancer. In this case, we were able to treat both the AL and the EPF with the EVT using the Eso-Sponge®. This rare, life-threatening complication was successfully treated without surgical intervention. Its management is challenging, controversial, and lacks an international consensus.

## Statement of Ethics

An ethical approval was not required. EVT is an established modality for the treatment of ALs after esophagectomy. A written informed consent was obtained from the patient for publication of this case report with any accompanying images.

## Conflict of Interest Statement

The authors have no conflicts of interest to declare.

## Funding Sources

No funding was provided for this case.

## Author Contributions

Imad Kamaleddine contributed to the drafting of the manuscript and revising the final draft; Imad Kamaleddine, Magdalena Popova, Ahmed Alwali, and Clemens Schafmayer contributed to the acquisition of data and revising the final draft; Imad Kamaleddine and Clemens Schafmayer contributed to the investigation and interpretation of the data; all authors have read and approved the manuscript.

## Data Availability Statement

All data generated or analyzed during this study are included in this article and its online supplementary material files. Further inquiries can be directed to the corresponding author.

## Supplementary Material

Supplementary data

Video 1 Supplemental Video

Video 2 Supplemental Video
