# Supplementary material for: Endoscopic Vacuum Therapy for Treating an Esophago-Pulmonary Fistula after Esophagectomy: A Case Report and Review of the Literature
Source: Visc Med. 2023 Feb 23;39(1):18–24. doi: 10.1159/000529725 (PMC10130739; doi:10.1159/000529725)
Supplement: Supplementary file 1 — Supplementary data [file vis-0039-0018-s01.pdf]

CARE case report guidelines

CARE Checklist of information to include when writing a case report

(cc) BY-NC-ND

Topic	Item	Checklist item description	Reported on Line
Title	1	The diagnosis or intervention of primary focus followed by the words "case report"	2
Key Words	2	2 to 5 key words that identify diagnoses or interventions in this case report, including "case report"	26
Abstract (no references)	3a	Introduction: What is unique about this case and what does it add to the scientific literature?	31-32
	3b	Main symptoms and/or important clinical findings	35
	3c	The main diagnoses, therapeutic interventions, and outcomes	35-36, 39-40
	3d	Conclusion—What is the main "take-away" lesson(s) from this case?	42-43
Introduction	4	One or two paragraphs summarizing why this case is unique (**may include references**)	55-58
Patient Information	5a	De-identified patient specific information.	60
	5b	Primary concerns and symptoms of the patient.	60-62
	5c	Medical, family, and psycho-social history including relevant genetic information	62-64
	5d	Relevant past interventions with outcomes	62-63
Clinical Findings	6	Describe significant physical examination (PE) and important clinical findings.	77-78, 103-104
Timeline	7	Historical and current information from this episode of care organized as a timeline	77
Diagnostic Assessment	8a	Diagnostic testing (such as PE, laboratory testing, imaging, surveys).	79-82
	8b	Diagnostic challenges (such as access to testing, financial, or cultural)	—
	8c	Diagnosis (including other diagnoses considered)	81-82, 104-106
	8d	Prognosis (such as staging in oncology) where applicable	—
Therapeutic Intervention	9a	Types of therapeutic intervention (such as pharmacologic, surgical, preventive, self-care)	94-96, 101-102, 117-119
	9b	Administration of therapeutic intervention (such as dosage, strength, duration)	94-96, 101-102, 117-119
	9c	Changes in therapeutic intervention (with rationale)	—
Follow-up and Outcomes	10a	Clinician and patient-assessed outcomes (if available)	122-123
	10b	Important follow-up diagnostic and other test results	123-127
	10c	Intervention adherence and tolerability (How was this assessed?)	119-122
	10d	Adverse and unanticipated events	—
Discussion	11a	A scientific discussion of the strengths AND limitations associated with this case report	129-154
	11b	Discussion of the relevant medical literature **with references**.	182-188
	11c	The scientific rationale for any conclusions (including assessment of possible causes)	155-165
	11d	The primary "take-away" lessons of this case report (without references) in a one paragraph conclusion	193-197
Patient Perspective	12	The patient should share their perspective in one to two paragraphs on the treatment(s) they received	190
Informed Consent	13	Did the patient give informed consent? Please provide if requested	Yes ☒ No ☐
